# Right ventricular morphology and function following stage I palliation with a modified Blalock–Taussig shunt versus a right ventricle-to-pulmonary artery conduit

**DOI:** 10.1093/ejcts/ezw227

**Published:** 2016-07-15

**Authors:** James Wong, Pablo Lamata, Rahul H. Rathod, Sophie Bertaud, Nathalie Dedieu, Hannah Bellsham-Revell, Kuberan Pushparajah, Reza Razavi, Tarique Hussain, Tobias Schaeffter, Andrew J. Powell, Tal Geva, Gerald F. Greil

**Affiliations:** a Department of Imaging Sciences, Kings College London, St Thomas’ Hospital, London, UK; b Boston Children's Hospital, Harvard Medical School, Boston, USA

**Keywords:** Hypoplastic left heart syndrome, Norwood procedure, Cardiac MRI

## Abstract

**OBJECTIVES:**

The Norwood procedure for hypoplastic left heart syndrome (HLHS) is performed either via a right ventricle-to-pulmonary artery (RVPA) conduit or a modified Blalock–Taussig (MBT) shunt. Cardiac magnetic resonance (CMR) data was used to assess the effects of the RVPA conduit on ventricular shape and function through a computational analysis of anatomy and assessment of indices of strain.

**METHODS:**

A retrospective analysis of 93 CMR scans of subjects with HLHS was performed (59 with MBT shunt, 34 with RVPA conduit), incorporating data at varying stages of surgery from two congenital centres. Longitudinal and short-axis cine images were used to create a computational cardiac atlas and assess global strain.

**RESULTS:**

Those receiving an RVPA conduit had significant differences (*P*< 0.0001) in the shape of the RV corresponding to increased ventricular dilatation (*P* = 0.001) and increased sphericity (*P* = 0.006). Differences were evident only following completion of stage II surgery. Despite preserved ejection fraction in both groups, functional strain in the RVPA conduit group compared with that in the MBT shunt group was reduced across multiple ventricular axes, including a reduced systolic longitudinal strain rate (*P*< 0.0001), reduced diastolic longitudinal strain rate (*P* = 0.0001) and reduced midventricular systolic circumferential strain (*P* < 0.0001).

**CONCLUSIONS:**

Computational modelling analysis reveals differences in ventricular remodelling in patients with HLHS undergoing an RVPA conduit insertion with focal scarring and volume loading leading to decreased functional markers of strain. The need for continued surveillance is warranted, as deleterious effects may not become apparent until later years.

## INTRODUCTION

The Norwood procedure has been the mainstay of surgical palliation for hypoplastic left heart syndrome (HLHS) over the last 30 years [1]. Soon after birth patients undergo surgical creation of a neoaorta, resulting in a systemic right ventricle (RV). Blood flow to the lungs is provided by insertion of a systemic-to-pulmonary shunt (stage I palliation). Subsequent stages of surgery at ages 4–6 months and 2–4 years seek to maintain pulmonary perfusion by diversion of blood from the superior vena cava (stage II) and then blood from the inferior vena cava (IVC) (stage III) directly to the pulmonary arteries via a cavopulmonary anastomosis.

There are two different types of systemic-to-pulmonary artery shunts. The modified Blalock–Taussig (MBT) shunt involves a connection between the innominate or subclavian artery and one of the branch pulmonary arteries. In some studies, the MBT shunt has been associated with haemodynamic instability [2] and sudden death [3] in the postoperative period. The right ventricle-to-pulmonary artery (RVPA) conduit was developed to offset this risk [4]. Following a ventriculotomy, a conduit is inserted between the RV and the main pulmonary artery. This results in volume loading from conduit regurgitation and a scar on the systemic ventricle predisposing the patient to an increased risk of arrhythmias or aneurysmal dilatation of the outflow tract [5]. Recent evidence from a large randomized trial initially showed no differences in echocardiographic measures of RV ejection fraction (EF) at 1 year of age [6]. In the follow-up study of the same cohort, RVEF had deteriorated significantly in the RVPA conduit group and transplant-free survival rates at 32 months of age were indistinguishable between the two shunt strategies [7].

Evidence is beginning to point towards poorer long-term outcomes for those receiving the RVPA conduit. At present, the direct impact of the ventriculotomy scar on the growth and motion of the systemic RV remains incompletely understood [8]. Cardiac magnetic resonance (CMR) provides detailed three-dimensional (3D) images of the RV, but applying traditional two-dimensional (2D) measurements such as length and diameter to assess a complex 3D shape does not capture subtle variations in topology. Computational anatomical analysis of CMR images permits the detection of small differences in ventricular geometry through the creation of detailed 3D cardiac atlases [9, 10]. In this study, we used this technique to examine the impact of the RVPA conduit on the growth and function of the systemic RV in patients with HLHS.

## METHODS

### Study population

We conducted a retrospective analysis on CMR datasets from two congenital cardiac centres. CMR scans of patients who underwent RVPA conduit insertion were provided by Boston Children's Hospital, USA (ethical approval IRB-P00012488). CMR scans of patients who underwent MBT shunt insertion were provided by Evelina London Children's Hospital, UK (ethical approval 08/H0810/058). Datasets consisted of balanced steady-state free precession cine imaging in four-chamber and short-axis orientations acquired on a Philips Achieva MRI scanner according to recent guidelines [11]. The most recent scans from both institutions from 2004 to 2014 of any patient with HLHS who underwent a Norwood procedure as the primary palliation were included and analysis occurred in a core laboratory (Kings' College London). At Evelina London Children's Hospital, all children with HLHS undergo routine preoperative CMR to help with surgical planning for stages II and III. Subjects only undergo cardiac catheterization if additional information is needed for planning or optimizing clinical status prior to surgery. At Boston Children's Hospital, CMR is performed on those considered lowest risk (i.e. no echocardiographic evidence of ventricular dysfunction, severe atrio-ventricular valve regurgitation, atrial restriction or arch obstruction), with most patients still also undergoing additional cardiac catheterization. Exclusion criteria for the study were patients with other diagnoses such as unbalanced atrioventricular canal defect, or those who received an initial procedure other than a Norwood procedure.

RV volumes and wall mass were measured using a Viewforum workstation (Viewforum, release 2.0, Philips Healthcare, Netherlands). End-diastolic volume (EDV) and end-systolic volume (ESV) were manually contoured from a cine short-axis stack corresponding to the ventricular cavity. Trabeculations were excluded from the blood volume [11]. The difference between EDV and ESV was the stroke volume (SV). EF was calculated as (EDV − ESV)/EDV × 100. Tricuspid regurgitation was recorded from the original MRI report (mild <15%, moderate 15–25%, moderate–severe 25–45% and severe >45%). The LV shape was recorded as globular, slit-like or borderline, as this was more likely to influence RV shape than the presence/absence of forward flow across the valves. Determination of Interuser variability was determined by James Wong and Kuberan Pushparajah.

### Creation of a cardiac atlas

**Figure 1:**
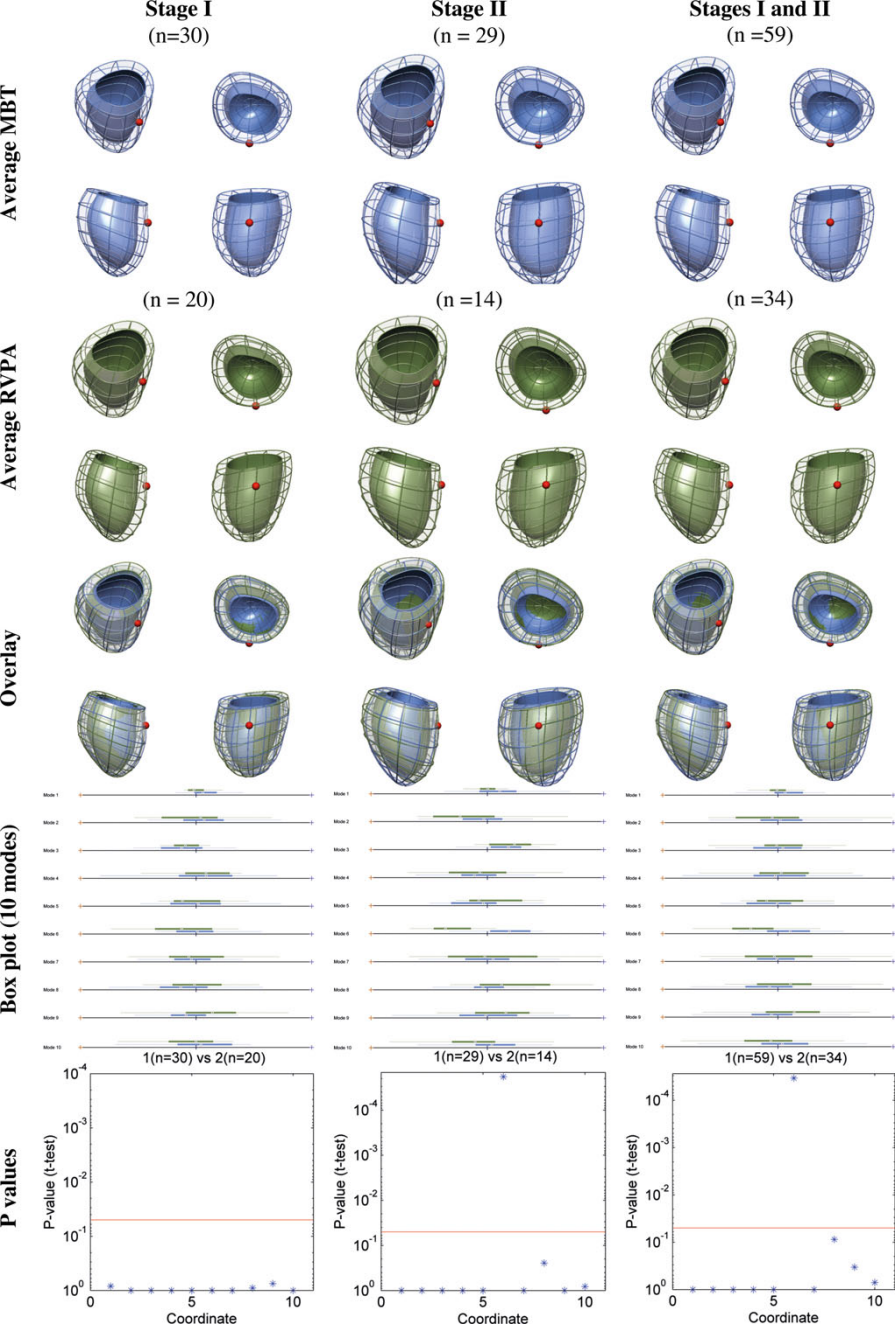
Ventricular shape differences between the RVPA conduit and MBT shunt groups. The top row shows the average ventricular shape for MBT shunts at each surgical stage and then an overall ventricular shape for both stages. The second row shows the equivalent average ventricular shape for RVPA conduits. The third row shows an overlay of the two averaged shapes for the MBT shunt and RVPA conduit groups. The solid colour represents the endocardial surface, while the mesh represents the epicardial surface. The position of the LV remnant is marked with a red sphere. Box plots for each of the first 10 cardiac atlases (Modes) are shown in the fourth row, with their corresponding *P*-values in the fifth row, showing a significant difference in shape in Mode 6 occurring only at stage II (*P*-values after Bonferroni correction; red line indicates the *P* = 0.05 threshold of significance). RVPA: right ventricle-to-pulmonary artery; MBT: modified Blalock–Taussig; LV: left ventricle.

The RV myocardium of all short-axis cine stacks was manually contoured at end-diastole, using ITK-SNAP (2.4.0) [12]. The septal wall was included as part of the RV, whereas the LV free wall was excluded from the segmentation because only the RV shape was of interest. Trabeculations were also excluded from the segmentation [11]. The position of the LV was marked on the most basal slice, as illustrated in Fig. 1, to correctly align each segmentation and allow comparison of cases.

A 3D geometrical model was then built from each RV segmentation using previously described methods [13]. The 3D geometrical model is described by a detailed set of 3456 variables that govern the geometry and orientation of the model in 3D space. They include traditional 2D geometric indices of thickness, length and diameter which were automatically extracted from these computational models.

Finally, a computational atlas was built from the collection of 93 RVs that described the change of shape from an average anatomy. To more easily permit comparison between the MBT and RVPA groups, the cardiac atlases that contained the largest variability in shape were detected using a principal component analysis. As a result, the description of each shape was reduced from 3456 variables to 10 variables. The variables that represent the largest variability in shape have previously been described in the literature as ‘anatomical Modes’ [13].

### Functional assessment

Longitudinal and short-axis cine images were used to compare systolic and diastolic strain in the two HLHS populations using CMR feature tracking software (Diogenes MRI, Tomtec, Germany). The endocardial borders of the RV were manually defined in one time frame and then automatically propagated through all time frames, tracking the endocardial–blood border and adjacent features to provide strain data. The four-chamber view was used to measure RV longitudinal function. The short-axis images were used to assess RV circumferential and radial function at basal, midventricular and apical levels. Short-axis stacks were linked to the position in the four-chamber view to choose the appropriate slice. Global strain was recorded as the mean of the segmental strain values for systolic strain, systolic strain rate and diastolic strain rates. Strain measures the change in length of the myocardium relative to its original length. Positive strain values indicate a lengthening of the ventricle (as seen in radial motion) and negative values indicate a shortening of the ventricle (as seen in longitudinal and circumferential motion). Values of a greater magnitude, irrespective of the vector, reflect improved motion.

### Statistical analysis

Statistical analysis was performed using SPSS version 22. Tests of normality were performed using the Shapiro–Wilk method. Continuous variables were expressed as mean ± SD. Comparisons between the MBT shunt and RVPA conduit groups for continuous variables were performed by unpaired *t*-tests, and for categorical variables by *χ*^2^ tests (e.g. tricuspid regurgitation and LV morphology). For analysing the cardiac atlases, which comprised 10 subgroups, one-way analysis of variance (ANOVA) with Bonferroni-adjusted, *post hoc t*-tests were used, as assumptions for ANOVA were met. Throughout a *P-*value of <0.05 was considered significant.

The Haycock method of calculating the body surface area (BSA) was used as an index to compare differently sized children. However, several studies have shown that there exists a non-linear relationship between cardiovascular dimensions and BSA within the paediatric population. The heart grows at a rate different from that of the body. This allometric growth of the heart means comparisons between individuals of different ages are best performed by indexing volumes to BSA raised to the power of 1.3 (ml/BSA^1.3^) and indexing measures of length to the square root of BSA (mm/√m^2^) [14, 15]. Indexing in this way has been shown to have a bearing on prognosis [16].

Interuser variabilities for manual segmentation of CMR were determined by intraclass correlation coefficients using a two-way random model with absolute agreement.

## RESULTS

There were a total of 93 CMR datasets from the two congenital centres. Of the total, 34 underwent RVPA conduit and 59 underwent MBT shunt insertion. The MBT shunt population comprised 30 stage I scans and 29 stage II scans. The RVPA conduit population comprised 20 stage I scans and 14 stage II scans. Demographic data are given in Table 1. A more limited ventriculotomy scar was introduced in those undergoing RVPA conduit insertion after 2009. Five stage I scans and three stage II scans had the more limited incision.

**Table 1:** Demographic data showing means and standard deviations

	MBT shunt	RVPA conduit	*P-*value
Number	59	34	
Birthweight	3.2 ± 0.4 kg	3.0 ± 0.5 kg	0.22
Re-coarctation	4/59	7/34	0.06
Occlusion of collateral vessels	8/59	33/34	**0.0001**
Stage 1 scans	30	20	
Age at stage I	7.1 ± 6.7 days	5.3 ± 2.8 days	0.28
Age at MRI scan	103 ± 24 days	141 ± 57 days	**0**.**0007**
BSA	0.40 ± 0.04 m^2^	0.44 ± 0.06 m^2^	**0**.**0087**
Stage II scans	29	14	
Age at Norwood	7.1 ± 6.7 days	7.3 ± 3.8 days	0.98
Age at stage II	172 ± 38 days	177 ± 65 days	0.72
Age at MRI scan	30.6 ± 8.2 months	27 ± 11 months	0.16
BSA	0.67 ± 0.23 m^2^	0.70 ± 0.11 m^2^	0.53

Values in bold indicate where *P* < 0.05.

MBT: modified Blalock–Taussig; MRI: magnetic resonance imaging; RVPA: right ventricle-to-pulmonary artery; BSA: body surface area.

There were no significant differences in birth weight or age at Norwood procedure between groups. Stage I scans for the RVPA conduit group tended to be performed later than for the MBT shunt group and, as expected, these patients were larger in size. Table 2 presents the MRI-derived data. Compared with the MBT shunt group, patients with an RVPA conduit had more dilated hearts at all stages of surgery (stage I, *P*= 0.001; stage II, *P* = 0.014). Indexed end-diastolic volume decreased following volume off-loading stage II surgery in both groups. There were no differences in tricuspid regurgitation. There were no differences in the range of LV morphology between the two groups.

**Table 2:** MRI-derived data for ventricular global function of the systemic right ventricle following either a modified Blalock–Taussig shunt or a right ventricle-to-pulmonary artery conduit

	MBT shunt	RVPA conduit	*P-*value
Total	*n* = 59	*n* = 34	
iEDV ml/ BSA^1.3^	80 ± 20	106 ± 33	**0**.**0001**
iESV ml/ BSA^1.3^	34 ± 13	47 ± 24	**0**.**001**
iSV ml/ BSA^1.3^	46 ± 11	59 ± 15	**0**.**0001**
EF %	57.9 ± 8.3	56.5 ± 8.5	0.43
Stage 1	*n* = 30	*n* = 20	
iEDV ml/ BSA^1.3^	83 ± 23	110 ± 32	**0**.**001**
iESV ml/ BSA^1.3^	38 ± 15	48 ± 24	0.087
iSV ml/ BSA^1.3^	44 ± 10	62 ± 13	**0**.**0001**
EF %	55.1 ± 7.6	58.3 ± 9.1%	0.18
TR			
None	14	9	0.75
Mild	13	8	
Moderate	3	3	
Severe	0	1	
LV shape			
Globular	17	17	0.92
Slit-like	10	8	
Borderline	3	3	
Stage II	*n* = 29	*n* = 14	
iEDV ml/ BSA^1.3^	77 ± 17	98 ± 33	**0**.**014**
iESV ml/ BSA^1.3^	30 ± 9	47 ± 19	**0**.**001**
iSV ml/ BSA^1.3^	47 ± 13	51 ± 17	0.38
EF %	60.6 ± 8.7	53.4 ± 6.6	**0**.**006**
TR			
None	17	6	0.31
Mild	10	7	
Moderate	2	0	
Severe	0	1	
LV shape			
Globular	18	9	0.30
Slit-like	8	2	
Borderline	2	3	

Values are indexed to BSA. Values in bold indicate where *P* < 0.05.

SV: stroke volume; BSA: body surface area; MBT: modified Blalock–Taussig; RVPA: right ventricle-to-pulmonary artery; EF: ejection fraction; LV: left ventricle; iEDV: indexed end diastolic volume; iESV: indexed end systolic volume; iSV: indexed stroke volume; TR: tricuspid regurgitation.

### Geometric analysis

All 93 computational shapes were successfully created reaching a sub-voxel accuracy (average fitting error 1.43 mm). Average cardiac shapes for the MBT shunt and RVPA conduit groups at each stage are shown in Fig. 1.

**Figure 2:**
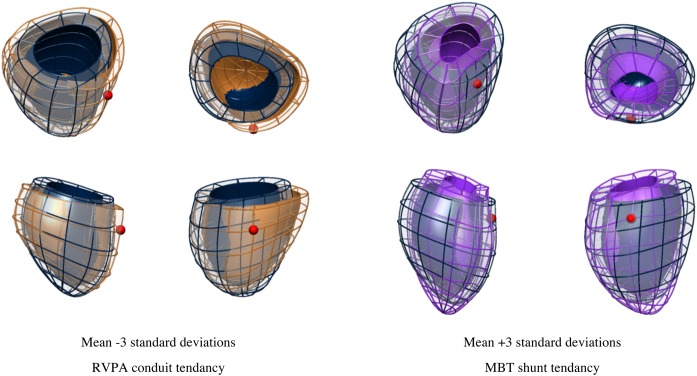
Changes in ventricular shape described by the anatomical Mode 6 are shown. Dark blue mesh represents the average ventricle of the 93 cases, and the brown and purple meshes illustrate the ±3 SDs of the range in shape variation. MBT shunt shows a positive (brown) tendency, and the RVPA conduit, a negative (purple) tendency. The shell represents the endocardial surface and the mesh is the epicardial surface. The position of the LV remnant is marked with a small red sphere. RVPA: right ventricle-to-pulmonary artery; MBT: modified Blalock–Taussig; LV: left ventricle.

The statistical atlas was built, and 10 cardiac atlases representing 79.0% of the variance in shape within the population were selected for analysis. A comparison between all RVPA and MBT groups revealed a significant difference in the mean of Mode 6 (*P* < 0.001; Fig. 1). A statistical difference in Mode 6 was not apparent between a comparison of RVPA versus MBT groups after stage I surgery, but then became statistically significant following stage II surgery (*P* < 0.001). This difference corresponded to the removal of the conduit. Figure 2 shows a visualization of the extreme shape variations (i.e. ±3 SDs) for Mode 6. The traditional geometrical measures in Table 3 revealed that the RVPA conduit group had a more spherical-shaped heart (*P*= 0.007), increased indexed RV cavity diameter (*P*= 0.006) and a larger indexed EDV (*P* < 0.0001, Table 2). However, the models in Fig. 2 additionally demonstrated subtle differences in size at the base of the ventricle and orientation of the apex. These topological differences were not captured by the parameters given in Table 3.

**Table 3:** Two-dimensional traditional geometrical measurements of the systemic right ventricle derived from personalized computational meshes, following either a modified Blalock–Taussig shunt or a right ventricle-to-pulmonary artery conduit

		MBT shunt	RVPA conduit	*P-*value
Total				
Myocardial mass	g/BSA^1.3^	104.4 ± 31.0	91.6 ± 50.5	0.12
Length	mm/√m^2^	77.8 ± 10.1	78.3 ± 14.2	0.82
Maximum ventricular cavity diameter	mm/√m^2^	53.0 ± 8.9	59.2 ± 13.3	**0**.**006**
Maximum ventricular epicardial diameter	mm/√m^2^	71.4 ± 7.2	74.5 ± 15.3	0.17
Sphericity ratio		1.49 ± 0.25	1.35 ± 0.25	**0**.**007**
Maximum wall thickness	mm/√m^2^	8.9 ± 2.4	7.9 ± 2.8	**0**.**044**
Standard deviation of septal wall thickness	mm/√m^2^	2.45 ± 0.76	2.76 ± 1.06	0.10
Stage I				
Myocardial mass	g/√m^2^	115.9 ± 32.1	99.3 ± 56.0	0.17
Length	mm/√m^2^	81.0 ± 10.5	78.1 ± 11.3	0.31
Maximum ventricular cavity diameter	mm/√m^2^	55.4 ± 7.6	56.6 ± 10.9	0.63
Maximum ventricular epicardial diameter	mm/√m^2^	74.8 ± 6.5	70.3 ± 7.4	**0**.**020**
Sphericity ratio		1.47 ± 0.15	1.41 ± 0.28	0.29
Maximum wall thickness	mm/√m^2^	9.4 ± 2.4	7.8 ± 2.9	**0**.**021**
Standard deviation of septal wall thickness	mm/√m^2^	2.67 ± 0.86	2.65 ± 1.03	0.94
Stage II				
Myocardial mass	g/√m^2^	91.7 ± 24.6	77.2 ± 33.7	0.11
Length	mm/√m^2^	74.2 ± 8.6	78.9 ± 18.9	0.27
Maximum ventricular cavity diameter	mm/√m^2^	50.3 ± 9.6	64.0 ± 16.3	**0**.**001**
Maximum ventricular epicardial diameter	mm/√m^2^	67.5 ± 5.8	81.2 ± 21.6	**0**.**003**
Sphericity ratio		1.52 ± 0.35	1.24 ± 0.19	**0**.**006**
Maximum wall thickness	mm/√m^2^	8.4 ± 2.4	8.0 ± 2.8	0.64
Standard deviation of septal wall thickness	mm/√m^2^	2.24 ± 0.56	2.97 ± 1.13	**0**.**006**

Values show the means and standard deviations and are indexed to the BSA. Values in bold indicate where *P* < 0.05.

BSA: body surface area; MBT: modified Blalock–Taussig; RVPA: right ventricle-to-pulmonary artery.

### Functional analysis

There were no differences in global systolic function between the MBT shunt and the RVPA conduit groups. Initially, EF was equivalent between groups at stage I surgery, but after stage II surgery EF was significantly lower in those receiving a RVPA conduit (*P =* 0.014), although the mean value was within the normal range.

**Figure 3:**
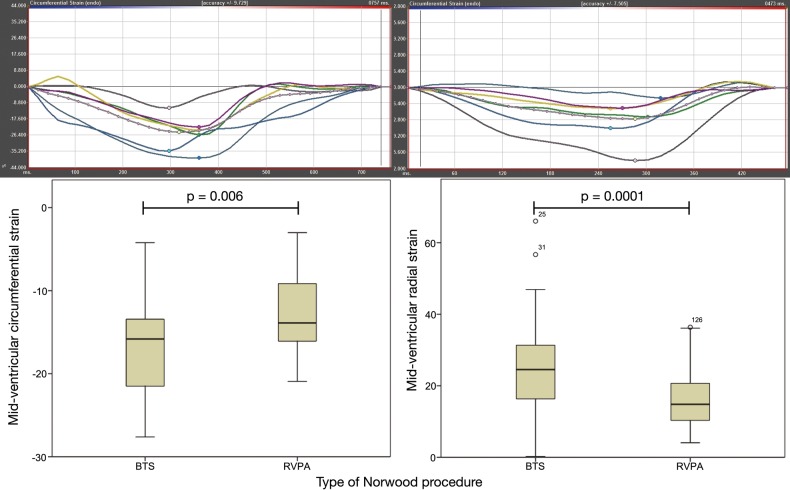
Feature tracking-derived MR strain. Top panel shows strain curves with a typical circumferential strain for a patient with the MBT shunt on the left and a patient with the RVPA conduit on the right. Segmental analysis is represented by each colour and white represents the mean global strain. The bottom panel shows a box and whisker plot comparing circumferential strain (left) and radial strain (right) for MBT shunts versus RVPA conduits. Circumferential strain is a negative value representing a shortening, while radial strain is a positive value representing a lengthening. MR: magnetic resonance; MBT: modified Blalock–Taussig; RVPA: right ventricle-to-pulmonary artery.

Table 4 gives the results of the strain analysis using feature tracking. Compared with those from the MBT shunt group, subjects from the RVPA conduit group had reduced longitudinal systolic function (strain *P* = 0.023, strain rate *P* < 0.0001). Furthermore, at the midventricular level, the RVPA conduit group displayed reduced circumferential strain (*P* < 0.006) and radial strain (*P* < 0.0001). Diastolic strain rates in the RVPA conduit group were reduced on assessment of longitudinal function (strain rate *P* < 0.0001), midventricular radial function (strain rate *P* = 0.011) and apical radial function (*P* = 0.029). Typical strain curves are shown in Fig. 3.

**Table 4:** Systolic and diastolic function of the systemic right ventricle following either a modified Blalock–Taussig shunt or a right ventricle-to-pulmonary artery conduit

	MBT shunt	RVPA conduit	*P-*value
Ejection fraction %	57.9 ± 8.3	56.5 ± 8.5	0.44
Systolic function			
Longitudinal			
Strain %	−14.78 ± 5.68	−12.23 ± 4.52	**0**.**023**
Strain rate % s^−1^	−1.37 ± 0.61	−0.92 ± 0.28	**0**.**0001**
Circumferential			
Basal strain %	−12.53 ± 4.80	−10.44 ± 3.56	0.15
Basal strain rate % s^−1^	−0.82 ± 0.34	−0.73 ± 0.22	0.19
Mid-strain %	−16.90 ± 5.14	−12.97 ± 4.63	**0**.**006**
Mid-strain rate % s^−1^	−1.17 ± 1.11	−0.94 ± 0.34	0.22
Apical strain %	−17.28 ± 9.54	−18.03 ± 6.98	0.070
Apical strain rate % s^−1^	−1.30 ± 0.84	−1.38 ± 0.50	0.35
Radial			
Basal strain %	16.11 ± 12.45	15.82 ± 7.47	0.38
Basal strain rate % s^−1^	1.16 ± 0.52	1.10 ± 0.41	0.25
Mid-strain %	24.76 ± 11.89	16.22 ± 8.05	**0**.**0001**
Mid-strain rate % s^−1^	1.32 ± 0.69	1.17 ± 0.44	0.14
Apical strain %	11.08 ± 8.32	9.95 ± 7.72	0.15
Apical strain rate % s^−1^	1.31 ± 0.78	1.14 ± 0.75	0.12
Diastolic function			
Longitudinal			
Strain rate % s^−1^	1.82 ± 0.91	1.32 ± 0.61	**0**.**0001**
Circumferential			
Basal strain rate % s^−1^	1.04 ± 0.40	0.99 ± 0.36	0.63
Mid-strain rate % s^−1^	1.46 ± 0.52	1.27 ± 0.50	0.054
Apical strain rate % s^−1^	1.63 ± 1.10	1.94 ± 0.75	0.12
Radial			
Basal strain rate % s^−1^	−1.22 ± 0.59	−1.11 ± 0.49	0.11
Mid-strain rate % s^−1^	−1.74 ± 0.94	−1.39 ± 0.68	**0**.**011**
Apical strain rate % s^−1^	−1.42 ± 0.82	−1.24 ± 0.87	**0**.**029**

Values in bold indicate where *P* < 0.05.

MBT: modified Blalock–Taussig; RVPA: right ventricle-to-pulmonary artery.

### Reproducibility of ventricular volumes

Intraclass correlation (95% confidence interval) was 0.97 (0.80–0.99) for EDV, 0.95 (0.82–0.980) for ESV, 0.95 (0.82–0.98) for SV and 0.89 (0.58–0.97) for EF.

## DISCUSSION

Interest in outcomes following a RVPA conduit is due to concern about the impact of a residual scar at the site of the conduit insertion on the systemic RV. Postmortem studies have shown that the scar remains stable in size irrespective of patient age and consists of fibrosis and ventricle wall thinning [17]. Echocardiographic studies have found wall motion abnormalities localized to the ventriculotomy site [18], with altered contractility occurring only after removal of the conduit and formation of a scar. Recent studies have compared survival rates, rates of intervention and transplant-free survival between the two types of shunts used in stage I operations [6, 7]. Survival at 3 years was comparable, but longer term prognoses remain uncertain. We hypothesized that the focal scarring following insertion of a RVPA conduit would adversely affect the shape and motion of the RV and, through computational shape analysis and strain analysis of MRI data, we would be able to detect differences between the Norwood groups. The major results of this study demonstrated that those receiving a RVPA conduit tended to have a more spherical-shaped and dilated ventricle, and, despite preserved EF, had markedly reduced functional strain values.

The use of computational modelling [9] allowed the creation of a 3D computational atlas of ventricular anatomy, accounting for characteristic variations in the HLHS population. Creating a cardiac atlas has the advantage of not having to assume any typical shape or dimension. Instead, variations in a structure are described and analysed in 3D terms rather than being subject to the constraints of applying 2D measurements to try and describe a 3D space. This is illustrated in Fig. 2, where differences in the orientation of the apex and shape at the base of the ventricle are seen but not easily measured. Similar techniques have been used to study the LV in adults who were born prematurely [10], and we were able to apply this to accurately capture the geometry of the systemic RV. In this study, one of the 10 cardiac atlas modes captured the shape remodelling differences between the RVPA conduit and MBT shunt groups and corresponded to increased sphericity and dilatation of the heart. The formation of the fibrotic scar after stage II surgery might cause reduced regional growth in the scar region relative to the remote myocardial wall, as previously described in echocardiographic and postmortem studies [17], leading to the observed change in the shape of the ventricle. The effects of pulmonary regurgitation through a non-valved conduit may also contribute to this difference in shape.

Our results demonstrated that those receiving RVPA conduits had more dilated ventricles at stage I than those receiving MBT shunts. This finding differs from that reported in the literature using echocardiography [19] and may reflect the fact that CMR more accurately records ventricular volumes [20] without the echocardiographic limitations of acoustic windows. EF has a prognostic value in patients with HLHS, and a decline in EF has been shown in those with RVPA conduits compared with those with MBT shunts at 3 years of age [7]. We found that EF was lower in the RVPA conduit group compared with the MBT shunt group but still within the normal range. This finding might reflect the smaller sample size of this study but may also be influenced by the method of assessment, as there is increased variability in measurement between echocardiography techniques and the CMR used in this study [20]. Interestingly, despite the preserved EF, a comparison of feature tracking-based strain analysis between the two Norwood groups showed that the RVPA conduit group had significantly reduced strain in multiple ventricular axes. The CMR strain data are the first to be analysed for HLHS. As would be expected from the altered RV geometry, systemic RV strain values were markedly different from those of the healthy LV [10] and healthy RV [21]. Values were comparable to existing measures taken in those with HLHS via echocardiography [22]. The altered strain in the RVPA conduit group may represent dyskinesia caused by the scar, though increased volume loading from pulmonary regurgitation through the RVPA conduit may also play a role. Abnormal strain has been linked to an increased risk of adverse events in adults with LV dysfunction [23] and correlates to the risk of developing heart failure in adults with anatomically normal hearts. As such, strain analysis in the systemic RV may be a useful early marker of those who might have a poorer prognosis, particularly when the primary benefits of feature tracking are considered: It is able to rapidly extract measures of strain from conventional MR imaging techniques; it is not limited by acoustic windows; and it does not require new sequences or time-consuming post-processing.

## LIMITATIONS

Differences in clinical management certainly exist between the two groups because they were recruited from two centres. Some differences, such as the interventional coil occlusion rate, can be accounted for, but many subtle factors affecting the RV shape cannot be fully quantified. This may lead to centre-specific bias; however, single-centre recruitment would not remove this potential bias. The RVPA cohort undergoing primary CMR represented those with ‘best-physiology’, as patients with less than ideal physiology underwent primary cardiac catheterization. Despite this bias, marked differences in shape and function existed between the two groups after stage II surgery, with those undergoing BTS having better strain values across multiple axes.

The discrepancy in population size between the two groups is a consequence of the development of surgical techniques. The MBT shunt has been the standard approach for 30 years. The RVPA conduit has gained popularity during the past 10 years. Recent refinements, including a reduction in the size of the ventriculotomy, are becoming more commonplace. We advocate increasing the database size to include more subjects as they become available, allowing improved sensitivity to abnormalities. Further work would include an analysis of how the more recent limited ventriculotomy affects growth and function. Prospectively collected data on serial MRI scans from the same subject would make this a more powerful study.

Because patients receiving a RVPA conduit were older when they underwent stage I CMR, they experienced greater exposure to the effects of a higher Qp:Qs through the shunt. Qp:Qs was not routinely measured in either centre. Such measurements might have provided useful additional information on how the shunt affects ventricular volume.

There was no differences in the age at which each group under-went stage II surgery. There was increased ventricular dilatation in the RVPA group both at Stage I and Stage II scans. This indicates that a major proportion of the volume loading may be a consequence of the type and size of shunt rather than inter-stage duration.

The addition of catheterization pressure data would have provided further information on the loading effects on the ventricle. Unfortunately, not enough information was available to permit analysis.

The quality of the computational atlas is limited by the resolution of available images. Full 3D resolution images would provide greater anatomical detail. The automatical 3D mesh reconstruction of the systemic RV achieved a tolerable accuracy compared with the images of the adult healthy LV (average fitting error divided by average length: 1.43/78 mm vs 1.28/95 mm [10]), despite the greater difficulty for manual segmentation and automatic reconstruction of a structure with thinner walls and larger shape variability.

MRI feature tracking is susceptible to interuser variability, particularly for longitudinal and radial regional segmental assessments [24, 25]. Comparison of global circumferential strain with the gold standard of MR-tagged sequences does show good correlation. Regional strain analysis in the right ventricular outflow tract region would be desirable, but the current analytic technique does not provide reliable data and requires further refinement.

## CONCLUSION

Utilizing computational tools to create a cardiac atlas of ventricular geometry allows detection of subtle deviations in 3D shape that may not be adequately captured using 2D measurements. Alterations in the shape of the ventricle following RVPA conduit palliation are associated with abnormal function. Although the current evidence on short-term outcomes is equivocal, the results of this study warrant continued surveillance to assess the long-term outcomes in this vulnerable group of patients.

## Funding

This study has received funding by the Department of Health through the National Institute for Health Research (NIHR) comprehensive Biomedical Research Centre award to Guy's & St Thomas’ NHS Foundation Trust in partnership with King's College London and King's College Hospital NHS Foundation Trust and by Action Medical Research (grant code GN2401). Pablo Lamata holds a Sir Henry Dale Fellowship funded jointly by the Wellcome Trust and the Royal Society (grant no. 099973/Z/12/Z). The Division of Imaging Sciences receives support as the Centre of Excellence in Medical Engineering (funded by the Wellcome Trust and EPSRC; grant no. WT 088641/Z/09/Z) as well as the BHF Centre of Excellence (British Heart Foundation award RE/08/03). Nathalie Dedieu was funded through a BHF grant (PG/12/5/29350). The MRI scanner at King's College London is partly supported by Philips Healthcare. Rahul H. Rathod, Andrew J. Powell and Tal Geva were supported in part by the Higgins Family Non-invasive Cardiac Imaging Research Fund. Funding to pay the Open Access publication charges for this article was provided by the Wellcome Trust (grant no. 099973/Z/12/Z).

**Conflict of interest:** none declared.
